# Challenges in colorimetric evaluation of femtosecond laser cleaning on historical leather: influence of surface porosity and microstructure

**DOI:** 10.1038/s40494-026-02484-w

**Published:** 2026-05-08

**Authors:** Canan Yağmur Boynukara, Malte L. Welsch, Luca Zanotto, Laurie Caron, Mehmet Uguryol, Gurcan Mavili, Luca Razzari, Cyril Muehlethaler, Andreas Ruediger, Patrizio Antici

**Affiliations:** 1 INRS-EMT, 1650 Boul. Lionel-Boulet, Varennes, Québec J3X 1P7 Canada; 2 Dipartimento SBAI, Sapienza Università di Roma, Via A. Scarpa 14, 00161 Roma, Italia; 3 Department of Biochemistry, Chemistry, Physics and Forensic Science, University of Quebec at Trois-Rivières, 3351 Boul. des Forges, Trois-Rivières, QC G8Z 4M3 Canada; 4 Department of Conservation and Restoration of Cultural Property, Faculty of Architecture, Yıldız Technical University, 34349 Besiktas, Istanbul Türkiye; 5 Department of Traditional Turkish Arts, Mimar Sinan Fine Arts University, 34427 Fındıklı, Istanbul Türkiye

## Abstract

This study critically evaluates femtosecond (fs) laser cleaning of historical vegetable-tanned goat leather, addressing quantification on porous, heterogeneous surfaces. Artificially aged, graphite-contaminated samples were treated by fs-laser irradiation with varying fluence and shot number. Cleaning performance was assessed by high-resolution CIE L*a*b* colorimetry, complemented by quantitative porosity analysis using optical microscopy and ImageJ. Pore-size measurements showed a mixed distribution of small and large pores affecting cleaning uniformity. Fs-laser cleaning reduced visible contamination, with ΔE* approaching the perceptibility threshold of 2.0. However, the leather microstructure led to significant reflectance variability, underscoring the limitations of area-averaged colorimetry for cleaning assessment. Comparative measurements on graphite-contaminated parchment, with negligible porosity but pronounced micro-relief, showed that pore structure and micro-topography govern optical consistency. Overall, these findings underscore the need to interpret colorimetry in relation to surface morphology and support integrating spatially localized optical diagnostics into fs-laser cleaning protocols to preserve visual integrity.

## Introduction

Cleaning historical objects demands meticulous care and precision, as preserving the authenticity and integrity of their surfaces requires methods that minimize any risk of damage^1^. Consequently, the development of non-invasive, non-contact cleaning techniques has become crucial to ensuring both effective treatment and the long-term preservation of cultural artifacts.

Among these methods, nanosecond (ns) pulse lasers and femtosecond (fs) ultrashort-pulse lasers have recently gained significant attention due to their precision and commercial viability^2^. In this context, nanosecond (ns) systems are commonly referred to as short-pulse lasers, whereas femtosecond (fs) systems operate in the ultrashort-pulse regime. This distinction matters because pulse duration influences how energy is deposited in time and space, which in turn affects the balance between thermal and non-thermal interaction pathways during cleaning. Unlike conventional methods, laser cleaning operates as a non-contact process, utilizing parameters such as wavelength, pulse duration, and polarization to adapt to the specific needs of delicate materials. Its high degree of control not only reduces risks associated with mechanical or chemical cleaning approaches but also enhances efficiency and safety during treatment^3,4^. Furthermore, laser cleaning is often faster and more cost-effective, making it an appealing option for cultural heritage conservation^5^.

Initially applied for stone conservation, laser cleaning has since been extended to a wide range of heritage substrates, including metals, textiles, paintings, paper, and collagen-based materials, using nanosecond, picosecond, and femtosecond pulse regimes^6^. On structurally complex substrates such as textiles and other fibrous materials, surface texture and three-dimensional morphology can strongly influence both cleaning uniformity and the optical assessment of cleaning outcomes^7–9^.

Among these, paper artifact cleaning stands out as particularly challenging due to the need to preserve the delicate fibrous organic matrix^10^. Recognizing this complexity, numerous research groups^11–13^ have conducted extensive studies, achieving significant results that not only advance scientific understanding but also contribute to the development of safe and effective conservation practices. Similarly, parchment, a material historically used to transmit written and visual content across generations, is another delicate substrate derived from animal hide, closely related to leather, that has received considerable attention in laser cleaning research due to its fragility and historical significance^14–16^.

In femtosecond laser cleaning, the ultrashort pulse duration confines energy deposition to time scales shorter than the time required for significant heat diffusion, which can reduce the extent of collateral thermal loading compared to longer-pulse regimes. Depending on the optical properties of the contaminant and substrate at the irradiation wavelength, removal can proceed through selective absorption by the soiling layer, rapid localized heating and ejection of contaminant particles, and, in some cases, non-linear absorption processes at sufficiently high peak intensities. In conservation practice, these interaction pathways are exploited to maximize selectivity of removal while minimizing optical or structural alteration of the underlying material^11,12,17^.

Laser cleaning of collagen-based heritage materials has been investigated on parchment and leather in several studies, including work on historical leather that reported process optimization and cleaning assessment, underscoring both the potential of the approach and the need for careful parameter selection and diagnostic validation^18–23^.

Against this background, leather represents a particularly challenging substrate for both laser cleaning and colorimetric assessment, because its porous, morphologically heterogeneous surface can amplify reflectance variability and complicate quantitative evaluation^24,25^. Dust and soot deposition is a common conservation issue on historical leather and can contribute to both visual alteration and long-term degradation^26^. Because particulate soiling can lodge within microstructural recesses, its removal is often spatially non-uniform, which further complicates the interpretation of area-averaged optical metrics. In addition, pollutant gases in the presence of moisture can generate acidic species that oxidize surface dressings and penetrate the collagen network, leading to progressive darkening and accelerated deterioration^27^.

To contextualize the materials used in this study and to clarify the leather–parchment distinction relevant to collagen-based heritage substrates, we briefly summarize the traditional preparation background below.

Goat leather is the most widely utilized leather type in traditional bookbinding all over the world^1^. In classical Turkish bookbinding crafts, goat leather (sahtiyan) produced by vegetable tanning has also been extensively preferred for bookbindings and book covers over sheep, lamb, or cattle leather for centuries. This preference is due to its greater durability, uniform thickness, and superior workability compared to those other materials^28^. The first stage of processing animal skin involves cleaning and removing the hair. This stage is carried out through washing in baths with water usually including lime, which enables the hair removal, and followed by removing flesh, fat residues, and subcutis layer mechanically from the inner surface, a process known as “etleme” (fleshing) in Turkish practice^29,30^. It is important to wash the skin again with water to eliminate the residual lime or other depilatory materials.

Tanning is an important step that enhances leather’s durability and enables its use in various applications. Vegetable tannins have historically been the primary materials used for tanning leather^1^. For traditional vegetable tanning, the leather was soaked in baths containing extracts from tannin-rich plants such as nutgall and acorn hulls from valonia oaks, which were indispensable tanning agents for Ottoman tanners^31^. After tanning, the leather is dyed.

The leather samples used in this work were provided by a local craftsman and processed traditionally as described above. The parchment samples were supplied from a company (Kare Deri, Istanbul) manufacturing high-quality parchment through the following traditional methods: the goat skins were first washed to remove dirt acquired during slaughtering or transport, then underwent hair removal by treating with depilatory agents in large drums, followed by repeated washes to eliminate any residual depilatory agents, and “etleme” process described above. Finally, the wet skins were stretched onto steel frames and dried in heated cabinets without undergoing any tanning treatment. Any residues remaining after the “etleme” process were removed by gentle abrasion. This process yielded parchment rather than leather, preserving the characteristic untanned collagen structure.

These differences in processing and structure motivate our focus on how surface morphology and microstructure influence both cleaning outcomes and the reliability of colorimetric analysis of collagen-based heritage substrates.

In this study, we investigated the application of femtosecond laser cleaning on traditionally processed and artificially aged bookbinding leather contaminated with graphite powder. While laser cleaning was effective in visibly reducing surface soiling, significant variability was observed in post-cleaning colorimetric measurements. We hypothesize that this variation arises from the porous, uneven surface morphology of tanned leather, which affects laser-material interactions and reflectance. To explore this hypothesis further, additional tests were conducted on a graphite-contaminated parchment sample, which possesses a markedly smoother, less porous structure. Our findings suggest that surface morphology plays a critical role in determining the consistency and reliability of optical outcomes in fs-laser cleaning of collagen-based substrates.

## Methods

### Leather samples

For experimental use, both the vegetable-tanned goat leather and the parchment samples were treated similarly to replicate historical soiling and aging conditions. The leather samples were dyed with RODA Dye NF brand dyes in the colors Orange and Havana. These colors correspond to the color codes of RODA brand leather dyes, which were supplied by GMW, a German supplier that specializes in equipment and materials for paper conservators and bookbinders. To simulate soiling, graphite powder (AF spezial, Kropfmühl, D90 ≤ 25 µm) was applied to represent unburned carbon particles (soot). The D90 specification indicates that 90% of the graphite particles are smaller than 25 µm, reflecting the fine particulate nature of the contaminant and its potential to penetrate porous surface recesses. The leather samples, including both soiled and unsoiled specimens, were prepared following the aging conditions recommended in the BS EN ISO 17228:2015 standard. The aging process was conducted at a controlled temperature of 50 °C and a relative humidity of 90% for a duration of four days. This procedure ensured that the leather surfaces closely simulated aged and soiled conditions commonly observed in historical artifacts, providing a realistic context for laser cleaning experiments.

**Fig. 1:**
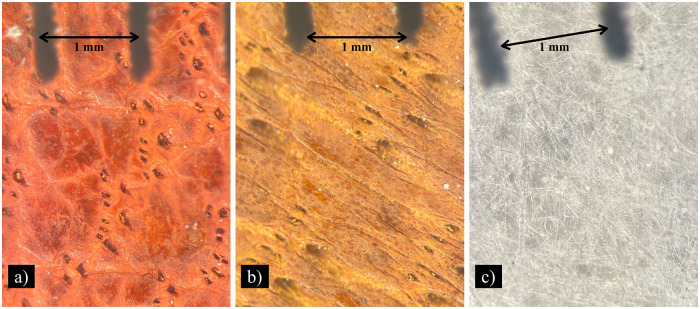
Surface morphology of representative samples captured at 2× magnification using an Olympus stereomicroscope. **a** Orange colored goat leather, **b** Havana colored goat leather, and **c** parchment. A 1 mm scale bar is shown in each image. The perceived colors in the images may slightly differ from the actual samples due to lighting and imaging parameters.

To provide visual context for the material surfaces after aging but prior to any soiling or laser treatment, low-magnification optical images were acquired using an Olympus stereomicroscope at 2× magnification. Figure 1 presents representative examples of (a) Orange colored leather, (b) Havana colored leather, and (c) parchment. A 1 mm scale bar is included in each image. These photographs are intended to give the reader a qualitative impression of the substrate appearance following artificial aging, helping to contextualize later observations on cleaning performance and material response.

**Fig. 2:**
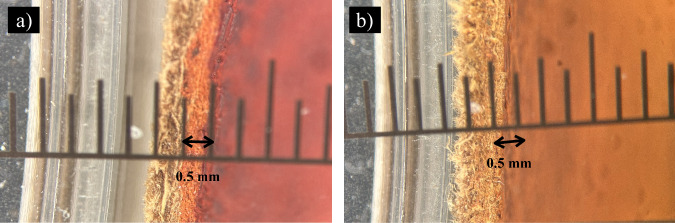
Cross-sectional morphology of dyed leather samples. Cross-sectional optical images of leather samples dyed with **a** Orange and **b** Havana RODA NF dyes. Images were acquired using an Olympus stereomicroscope at 2× magnification.

Cross-sectional microscopy was used to estimate the thickness of the dye layer. Scale-referenced optical images of the leather cross-sections, shown in Fig. 2a, b, were acquired using an Olympus stereomicroscope at 2× magnification. The dyed coatings were approximately 500 µm thick for both the Havana and Orange-colored samples. While the Havana dye appeared visually less distinct due to its similarity to the natural color of the leather substrate, its thickness was consistent with that of the orange dye, suggesting comparable dye penetration depth. These values confirm that the leather surface was uniformly dyed to a moderate depth, which may influence both laser–material interaction and post-cleaning optical behavior.

### Femtosecond laser cleaning setup

**Fig. 3:**
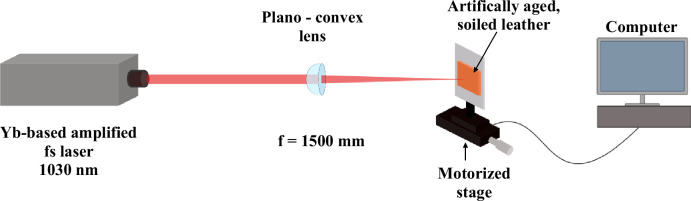
Schematic of the femtosecond laser cleaning setup.

The laser cleaning experiments were conducted using a Yb: KGW femtosecond laser amplifier (Pharos, Light Conversion), delivering pulses at a central wavelength of 1030 nm with a pulse duration of 170 fs. The laser beam was propagated through free space and focused onto the sample surface using a plano-convex lens (Thorlabs LA1254-B) with a focal length of 1500 mm, positioned at a working distance of approximately 1430 mm from the lens. The beam size at the sample position was measured using a beam-profiling camera and Gaussian fitting, yielding 1/*e*^2^ diameter values of *d*_*x*_ = 482 μm and *d*_*y*_ = 531 μm. The optical average power *P* of the laser beam was measured using a power meter (Ophir 3A-PF-12). The measured beam diameters and *P*, the peak fluence of an elliptical Gaussian beam, can be calculated as ${F}_{0}=\frac{2E}{\pi {w}_{x}{w}_{y}}$, where $E=\frac{P}{f}$ is the pulse energy, *f* is the laser repetition rate, while ${w}_{x}=\frac{{d}_{x}}{2}$ and ${w}_{y}=\frac{{d}_{y}}{2}$. A schematic illustration of the experimental configuration is shown in Fig. 3.

The samples were mounted vertically on a motorized translation stage (Thorlabs MTS50A-Z8), allowing fine positioning along the beam propagation direction to maintain focus. Experiments were performed at normal incidence in ambient air. Laser treatment was performed using translation-stage line-scanning along a single axis. The laser repetition rate was set to *f* = 100 Hz, and the exposure was applied as a single translation-stage line scan with a length of 10 mm. Two exposure conditions were implemented by changing the scan speed:${v}_{1}=1\,\mathrm{mm}/{\rm{s}}$ and ${v}_{2}=0.5\,\mathrm{mm}/{\rm{s}}$ corresponding to elapsed scan times of approximately 10 s and 20 s, respectively. The scan was computer-controlled, and the scan time was additionally monitored as a consistency check. Beam overlap along the scan direction was quantified using the pulse-to-pulse spacing $\Delta x=v/f$, giving 10 µm (1 mm/s) and 5 µm (0.5 mm/s). Based on the measured spot size, an effective beam diameter ${d}_{\mathrm{eff}}=\sqrt{{d}_{x}{d}_{y}}\approx 506\,\mathrm{\mu m}$ was used to estimate the effective pulse accumulation per surface location along the scan direction $N\approx f{d}_{\mathrm{eff}}/v$. With *f* = 100 Hz, the 1 mm/s condition yields $N\approx$ 50 pulses per beam diameter, while the 0.5 mm/s condition yields $N\approx$100 pulses per beam footprint; these settings are therefore referred to as the “50-shot” and “100-shot” conditions. Importantly, these shot numbers do not correspond to multiple pulses fired at a single spot, but to the effective pulse accumulation during continuous line scanning.

To investigate the influence of pulse energy and cumulative dose on cleaning performance, pulse energies were varied from 110 to 150 µJ for Orange colored leather and 90 to 120 µJ for Havana colored leather, corresponding to peak fluences of 0.11–0.15 J/cm^2^ and 0.0895–0.1194 J/cm^2^, respectively. For parchment, pulse energies of 120–180 µJ were explored, corresponding to 0.12–0.18 J/cm^2^. Each condition was tested under both scan settings corresponding to approximately 50 and 100 effective pulse accumulations per surface location along the scan direction to evaluate the effect of cumulative exposure on cleaning efficacy.

The applied energy ranges were determined based on preliminary screening tests. Microscopic inspection after laser exposure revealed that Havana colored leather could be overcleaned even at relatively low energies, whereas Orange colored leather required higher energy levels to exhibit measurable cleaning. Accordingly, different energy ranges were assigned to each leather type to ensure material-specific optimization, rather than direct comparison between them.

### Optical microscopy for surface morphology analysis

Optical microscopy was performed using a Zeiss Axio Scope A1 upright microscope (Carl Zeiss Microscopy GmbH, Carl-Zeiss-Promenade 10, 07745 Jena, Germany) equipped with a 10× objective lens. For each sample, images were collected from three different regions on clean (uncontaminated) but artificially aged leather substrates to assess surface morphology, pore distribution, and porosity parameters. Images were captured using ZEN 2 core software (version 2.5; Carl Zeiss Microscopy GmbH, Germany) under consistent illumination and contrast settings to ensure comparability across samples.

Porosity analysis was conducted using ImageJ software (NIH, USA). Scale calibration was performed by drawing a line along the 200 µm scale bar embedded in each image and using the *“Set Scale”* function to define the known distance (200 µm) with micrometers as the unit of length. Following calibration, pore areas were measured manually using the *Polygon Selection* tool. This tool was chosen because pores exhibited irregular shapes that could not be accurately captured with predefined circular or rectangular selections, allowing free-form outlining of each pore boundary to ensure precise area measurement. Each pore was individually outlined and measured via the *Analyze* *>* *Measure* function in ImageJ, with results recorded in the measurement table for subsequent analysis. Across the analyzed images, an average of approximately 10 pores per image (typically 5–12, depending on the region and material) were individually outlined and measured. Finally, the total image area was measured by selecting the entire image and using the *Analyze* *>* *Measure* function. Porosity (%) was calculated using the following Eq. (1):

1 $$Porosity\,\left( \% \right)=\frac{Total\,pore\,area}{Total\,image\,area}\times 100$$

where total pore area is the sum of all individually measured pore areas, and total image area is the area of the entire image field.

These procedures were repeated for Orange colored leather, Havana colored leather, and parchment samples. For each material, three separate images from different regions were analyzed to account for spatial variability in surface porosity. The porosity values obtained from these replicates were used to calculate the mean porosity and standard deviation for each material type, providing a quantitative estimate of both average porosity and its variability. It should be noted that this approach provides a two-dimensional (2D) estimation of porosity based on surface images and does not account for the full three-dimensional (3D) pore network within the material.

Pore size measurements were performed using ImageJ software after calibration with the known scale bar present in each optical microscopy image. Following scale calibration, the *Line* tool was used to draw a straight line across the maximum diameter of each visually identifiable pore, and the measured length was recorded in micrometers. This approach allowed for rapid estimation of pore diameters to complement the quantitative area-based porosity analysis.

### Color measurement

To evaluate the effectiveness of the laser cleaning process, color measurements were performed on laser-cleaned, artificially aged, soiled leather samples and compared with non-soiled, aged reference samples. The VSC® 8000/HS (Foster + Freeman, UK) was used for this purpose, utilizing the CIE L*a*b* color space to analyze the lightness (L*), green-to-red (a*), and blue-to-yellow (b*) components. The differences in these values, expressed as ΔL*, Δa*, and Δb*, were calculated to assess the relative color changes between the cleaned areas and reference samples.

Achieving an ΔE* value, which combines ΔL*, Δa*, and Δb* into a single metric, is defined in Eq. (2)^32^:

2 $$\Delta E* =\sqrt{{\left(\Delta L* \right)}^{2}+{\left(\Delta a* \right)}^{2}+{\left(\Delta b* \right)}^{2}}$$

Achieving a ΔE* value, which combines ΔL*, Δa*, and Δb* into a single metric as close to zero as possible, is crucial for maintaining the esthetic integrity of historical artifacts^32,33^. A Δ*E* value below ~2 is particularly desirable, as it is imperceptible to the human eye and indicates minimal visual alteration^34^. Ensuring minimal color change not only preserves the esthetic properties but also avoids chemical degradation, as discoloration often signifies underlying structural changes^35^.

For Havana colored leather, Orange colored leather, and parchment samples, optical microscope images were acquired from three different regions on clean (uncontaminated) but artificially aged samples to assess surface morphology and quantify porosity prior to laser exposure.

For each laser-treated area, L*, a*, and b* values were collected from three different positions within the ablation zone. The same procedure was applied to the corresponding reference area. Mean values and standard deviations were computed for each color coordinate in the treated group, while only the mean values were considered for the unsoiled reference.

Since the surface of artificially aged leather is inherently rough and morphologically irregular, uniform laser–material interaction across the entire irradiated area cannot be assumed. The presence of microtopographic features, such as pores, ridges, and depressions, alters the local angle of incidence and effective fluence, thereby affecting the energy absorbed at each point. Consequently, certain zones, such as elevated protrusions, may be cleaned more efficiently, while recessed or shadowed areas may remain partially soiled despite identical laser parameters. To capture this within-area variability, color measurements were therefore taken at three spatially separated points within each laser-treated zone. The points were selected to represent regions of higher, intermediate, and lower local micro-relief within the scanned area, thereby sampling more exposed surface features as well as more recessed zones where partial shielding can occur. Our spectrophotometer enabled spot-wise measurements on areas smaller than the 500 µm laser beam diameter, allowing reflectance data to be recorded from peak, valley, and intermediate zones across the cleaned area. The leather surface presents a heterogeneous micro-relief dominated by pores and fine fiber bundles, each with randomly oriented facets. In principle, such structures can lead to anisotropic or direction-dependent reflectance, particularly when measured under highly oblique illumination geometries. In this work, color was measured under a near-normal illumination/observation geometry (illumination angle ≤ 10° from the surface normal) using broadband visible illumination. At this spatial scale, corresponding to only a few pores per acquisition area, the reflected signal represents a local average of diffuse reflectance and is effectively insensitive to the sample orientation. Consequently, rotating the specimen would not alter the recorded CIE L*a*b* values beyond instrumental repeatability.

The total color difference (ΔE*) between the treated and untreated regions is given by Eq. 2, as described previously. To account for surface-related reflectance variability, the dispersion of L*, a*, and b* values across different points within the treated region was used to estimate the measurement uncertainty.

The combined uncertainty in ΔE* was estimated using first-order Gaussian error propagation. In this approach, *σ*_L*_, σ_a*_, and *σ*_b*_ denote the standard deviations of L*, a*, and b* across multiple measurement spots within the treated region, and covariance terms between L*, a*, and b* were neglected because of the limited number of spatial replicates. Consequently, *σ*_ΔE*_ should be interpreted as an approximate measure of spatial reflectance variability rather than as a rigorous statistical confidence interval. The following Eq. (3) was used:

3 $${\sigma }_{\Delta {E}^{* }}=\sqrt{{\left(\left(\Delta {L}^{* }\right)/\Delta {E}^{* }\right)}^{2}\cdot {\sigma }_{{L}^{* }}^{2}+{\left(\left(\Delta {a}^{* }\right)/\Delta {E}^{* }\right)}^{2}\cdot {\sigma }_{{a}^{* }}^{2}+{\left(\left(\Delta {b}^{* }\right)/\Delta {E}^{* }\right)}^{2}\cdot {\sigma }_{{b}^{* }}^{2}}$$

This yielded a single uncertainty value (±*σ*_ΔE*_) that reflects the spatial heterogeneity of the laser–material interaction, allowing for a more representative evaluation of cleaning performance. Given the limited number of spatial replicates, the reported standard deviations and *σ*_ΔE*_ are used here as indicators of within-area spatial variability rather than as statistically rigorous confidence intervals.

## Results

### Visual outcomes of femtosecond laser cleaning

**Fig. 4:**
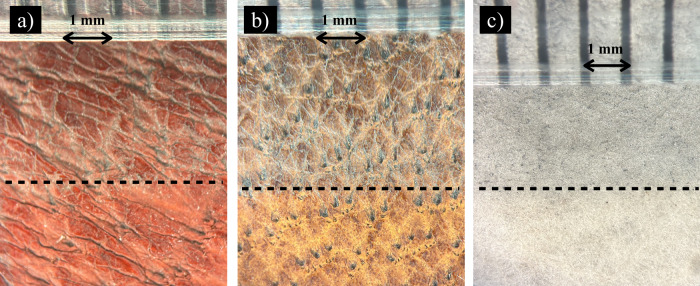
Surface images of the three sample types after contamination and aging process, acquired using an Olympus stereomicroscope at 2× magnification. Panels **a**–**c** correspond to Orange colored leather, Havana colored leather, and parchment, respectively. In each image, the upper region above the dashed black line shows the graphite-contaminated area, while the lower region corresponds to the laser-cleaned zone. The scale bar represents 1 mm. The perceived colors in the images may slightly differ from the actual samples due to lighting and imaging parameters.

Figure 4 presents representative stereomicroscope images of the three substrates after artificial soiling, aging, and partial fs-laser cleaning, illustrating the macroscopic visual outcome of the treatment. Panel (a) shows an Orange colored leather sample, panel (b) displays a Havana colored leather sample, and panel (c) corresponds to a parchment specimen. All samples were subjected to the same soiling and accelerated aging procedures prior to laser application. Under these conditions, laser irradiation visibly reduced surface soiling; however, the apparent uniformity of the cleaned zones differed between substrates. The images were acquired using an Olympus stereomicroscope at 2× magnification. In each case, the region above the black dashed line represents the graphite-contaminated surface, while the area below the line corresponds to the portion where fs-laser cleaning was applied. A 1 mm scale bar is included to provide spatial reference. This figure is intended to support visual interpretation of the cleaning effect and offers an illustrative comparison between soiled and laser-treated regions under optical magnification; it does not imply complete contaminant removal within the treated areas. Closer inspection of the treated zones suggests that darker residues can persist in pores and recessed micro-features, consistent with locally incomplete removal.

### Optical microscopy-based porosity comparison

**Fig. 5:**
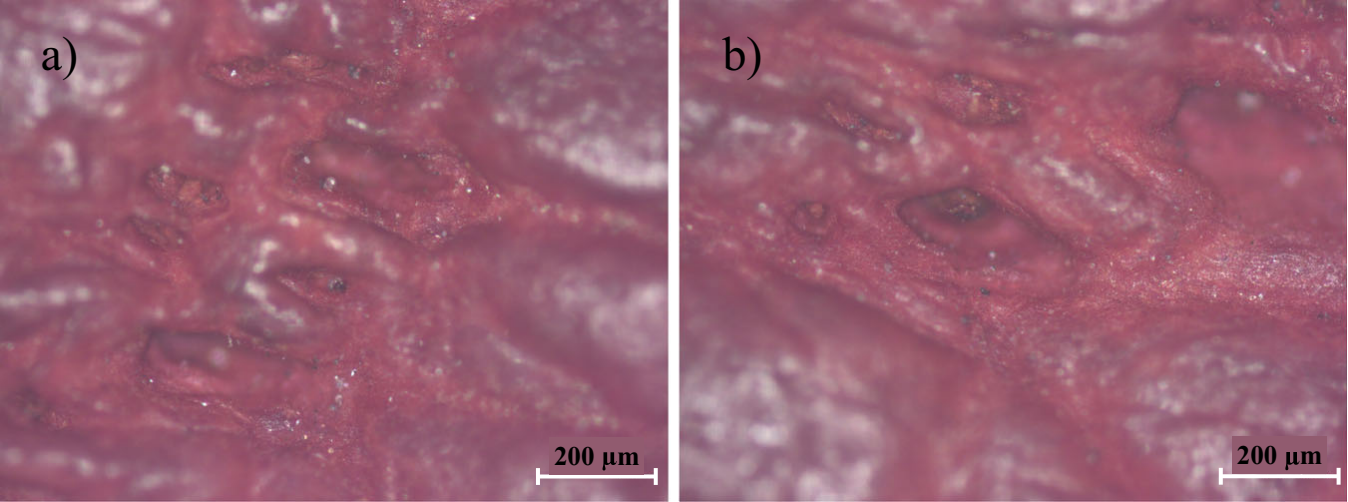
Optical microscopy images of Orange colored leather showing pore distribution at 10× magnification with a 200 µm scale bar. Images **a** and **b** were taken from different regions to assess spatial variability in surface porosity. The field of view for each image is approximately 1120 µm × 840 µm.

The surface porosity of Orange colored leather samples was evaluated using optical microscopy and ImageJ-based analysis. Representative images from two different regions are shown in Fig. 5, highlighting the spatial variability of pore distribution across the sample surface. Quantitative analysis of three replicate images yielded an average porosity of 7% and a standard deviation of 4%, indicating moderately low porosity and considerable heterogeneity across areas.

**Fig. 6:**
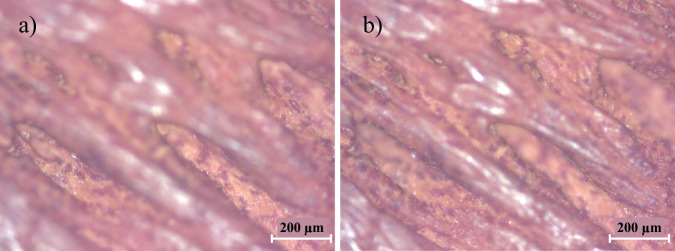
Optical microscopy images of Havana colored leather used for porosity analysis at 10× magnification with a 200 µm scale bar. Image **a** shows the general surface morphology, while image **b** illustrates a pore-focused view. The field of view for each image is approximately 1120 µm × 840 µm.

The surface porosity of Havana colored leather samples was assessed using optical microscopy and ImageJ-based analysis. Representative images are presented in Fig. 6, where (a) shows the general surface morphology and (b) illustrates a pore-focused view. Because the porous leather surface exhibits pronounced micro-relief, the limited depth of field at 10× magnification means that different features come into focus at different focal planes; therefore, panels (a) and (b) are shown at different focus settings to better visualize both the overall texture and the pore network. Quantitative analysis of three replicate regions yielded an average porosity of 14% and a standard deviation of 3%, indicating a moderately porous, heterogeneous surface.

**Fig. 7:**
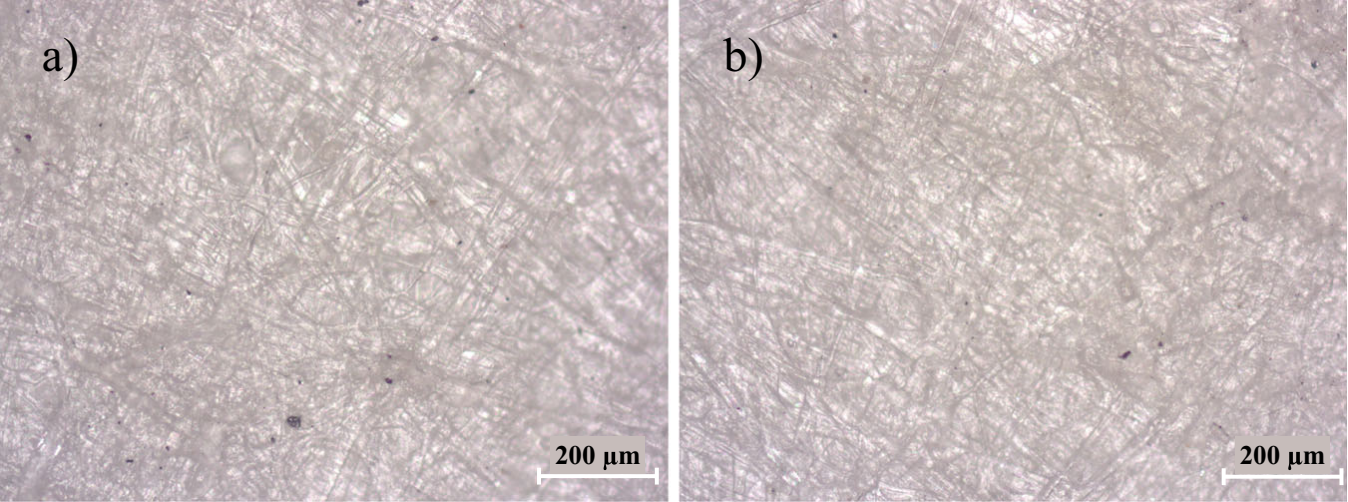
Optical microscopy images of a parchment sample showing pore distribution at 10× magnification with a 200 µm scale bar. Images **a** and **b** were taken from different regions to assess spatial variability in surface porosity. The field of view for each image is approximately 1120 µm × 840 µm.

Quantitative analysis of three replicate regions yielded an average porosity of 0.195% with a standard deviation of 0.165%, a value insignificant compared to the Orange and Havana colored leather samples. Representative optical microscopy images from two different regions are shown in Fig. 7. Overall, the parchment exhibited an almost pore-free structure, with only a few isolated pores detected across the analyzed areas. Such negligible porosity is consistent with the intended function of parchment, which was historically manufactured to provide a smooth, dense surface suitable for writing; the presence of pores would allow ink to penetrate and spread, compromising legibility. Nevertheless, despite its minimal pore content, the microstructure of parchment remains highly irregular, characterized by a complex landscape of peaks and valleys rather than a paper-like flatness, reflecting micro-topographical heterogeneity. This microstructural irregularity is expected to influence the colorimetric measurements discussed in the following sections.

**Fig. 8:**
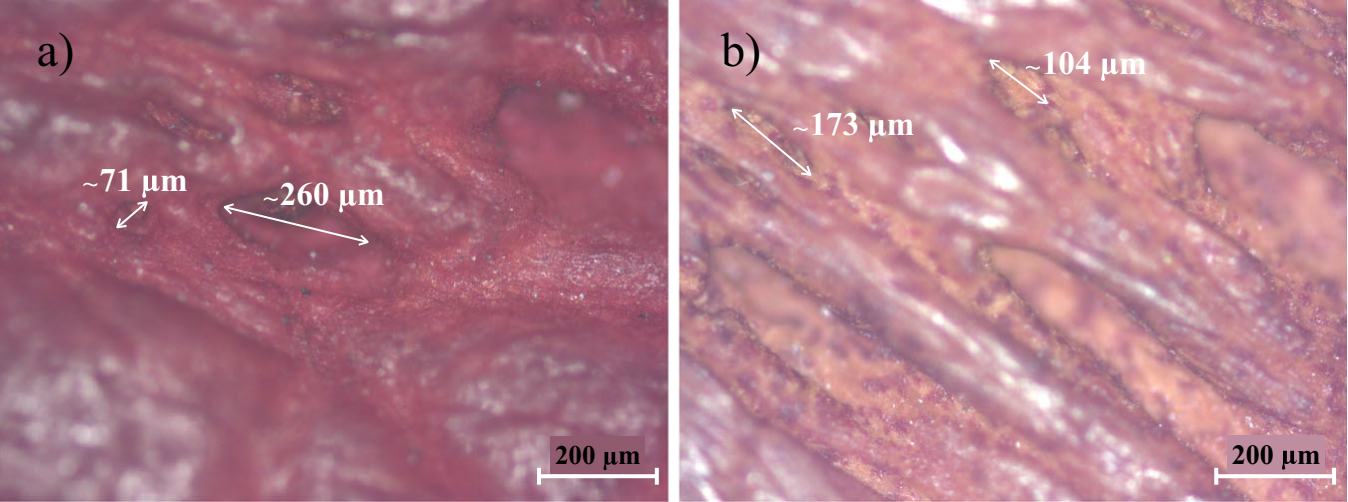
Representative pore size measurements from optical microscopy images: **a** Orange colored leather, **b** Havana colored leather. The field of view for each image is approximately 1120 µm × 840 µm. Approximate pore diameters are indicated in micrometers.

Pore size measurements were conducted on Orange and Havana colored leather samples to complement the porosity analysis. As shown in Fig. 8, both leathers exhibited a heterogeneous distribution of pore sizes, with diameters ranging from approximately 71 µm to 260 µm. This variability indicates that the surfaces contain both small and large pores, each influencing laser cleaning outcomes differently. If the same overall porosity were composed predominantly of numerous small pores, the surface would present less pronounced topographical variation, potentially enabling more uniform laser–material interaction and reduced reflectance variability. Conversely, if the porosity were concentrated in fewer but larger pores, shadowing effects and local fluence disparities would become more significant, leading to uneven cleaning and patchy visual results. The mixed pore size distribution observed in these samples likely amplifies reflectance variability and imposes inherent limitations on achieving consistent fs-laser cleaning performance.

### Colorimetry and reflectance variability

**Fig. 9:**
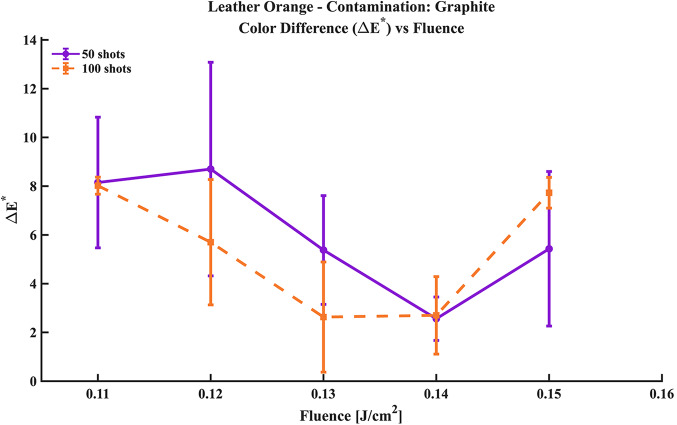
Color difference (ΔE*) values measured on an Orange colored leather sample contaminated with graphite after fs-laser cleaning, as a function of fluence for 50 and 100 laser shots. Error bars represent standard deviations from three replicate measurements.

As shown in Fig. 9 and Table 1, ΔE* values for the Orange colored leather sample exhibited a non-monotonic response to increasing fluence, particularly under 50-shot scanning conditions. The color difference ranged from 8.70 ± 4.38 to 2.56 ± 0.89, with the lowest value recorded around 0.14 J/cm^2^, indicating optimal cleaning performance at this fluence. At lower fluences, ΔE* values were elevated, likely due to incomplete removal of graphite residues. At higher fluences, such as 0.15 J/cm^2^, ΔE* values remained elevated: 5.43 ± 3.17 for 50 shots and 7.73 ± 0.63 for 100 shots. These results indicate the onset of overcleaning, in which not only residual graphite is removed but the dyed surface layer itself may be locally altered or partially ablated, resulting in changes in both lightness and chromatic coordinates. The large standard deviations observed across both lower and higher fluences, including 8.70 ± 4.38 at 0.12 J/cm^2^ and 5.43 ± 3.17 at 0.15 J/cm^2^, reflect notable variability in reflectance across the cleaned areas. This variability is likely caused by uneven laser–surface interaction, in which protruding regions received excessive exposure while recessed areas received insufficient exposure.

**Table. 1:** Total color difference ΔE* for the fs-laser cleaned Orange colored leather sample at varying fluence and shot numbers

Sample name	Fluence [J/cm^2^]	ΔE^*^ (50 shots)	ΔE^*^ (100 shots)
Orange colored	0.11	8.15 ± 2.68	8.02 ± 0.35
	0.12	8.70 ± 4.38	5.70 ± 2.57
	0.13	5.38 ± 2.23	2.63 ± 2.26
	0.14	2.56 ± 0.89	2.70 ± 1.59
	0.15	5.43 ± 3.17	7.73 ± 0.63

Values are reported as ΔE* ± propagated uncertainty (*σ*_ΔE*_).

In contrast, the 100-shot condition yielded a more consistent trend, with generally lower ΔE* values and a minimum of 2.63 ± 2.26 at 0.13 J/cm^2^. Although some variability persisted, the narrow error margins, such as 8.02 ± 0.35 at 0.11 J/cm^2^, indicate more uniform laser–material interaction. This supports the interpretation that increasing pulse count can mitigate the influence of microstructural irregularities, even though overcleaning remains a risk at higher fluences.

**Fig. 10:**
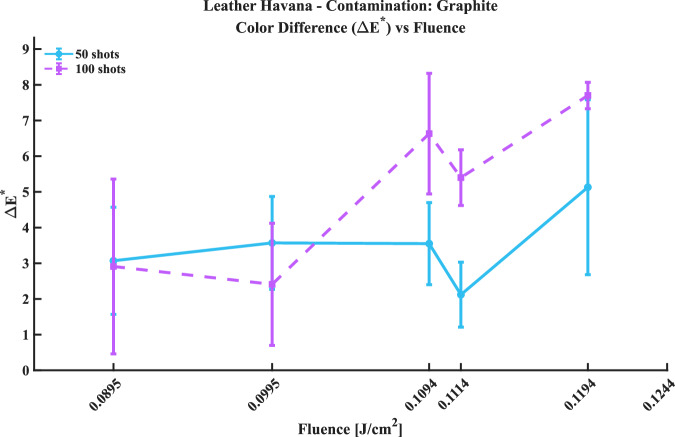
Color difference (ΔE*) values measured on Havana colored leather sample contaminated with graphite after fs-laser cleaning, as a function of fluence for 50 and 100 laser shots. Error bars represent standard deviations based on three replicate measurements.

As shown in Fig. 10 and Table 2, ΔE* values for the Havana colored leather sample remained relatively low and stable across the tested fluence range under 50-shot conditions. Between 0.0895 and 0.1114 J/cm^2^, ΔE* ranged from 3.07 ± 1.50 to 2.12 ± 0.91, without any significant spikes in variability. The lowest value was 0.1114 J/cm^2^, suggesting a favorable fluence window in which femtosecond laser interaction effectively removes graphite contamination while minimizing surface alteration. The narrow standard deviations observed throughout this range indicate a consistent and uniform cleaning response across the treated surface.

**Table. 2:** Total color difference ΔE* for the fs-laser cleaned Havana colored leather sample at varying fluence and shot numbers

Sample name	Fluence [J/cm^2^]	ΔE^*^ (50 shots)	ΔE^*^ (100 shots)
Havana colored	0.0895	3.07 ± 1.50	2.91 ± 2.45
	0.0995	3.57 ± 1.30	2.41 ± 1.71
	0.1094	3.55 ± 1.15	6.63 ± 1.69
	0.1114	2.12 ± 0.91	5.40 ± 0.78
	0.1194	5.13 ± 2.45	7.70 ± 0.37

Values are reported as ΔE* ± propagated uncertainty (*σ*_ΔE*_).

In contrast, the results obtained under 100-shot exposure revealed a distinct trend. At fluences below 0.0995 J/cm^2^, ΔE* values were relatively high and variable, pointing to insufficient cleaning. A clear minimum was observed at 0.0995 J/cm^2^, where ΔE* dropped to 2.41 ± 1.71, indicating optimal contaminant removal performance. However, beyond this point, the color difference increased rapidly, reaching 6.63 ± 1.69 at 0.1094 J/cm^2^ and 7.70 ± 0.37 at 0.1194 J/cm^2^. These elevated values likely result from overcleaning effects, where excessive fluence may cause undesirable changes to surface reflectance or fiber morphology. This behavior emphasizes that while extended exposure can enhance cleaning efficacy, it also narrows the safe processing window and increases the risk of esthetic degradation.

**Fig. 11:**
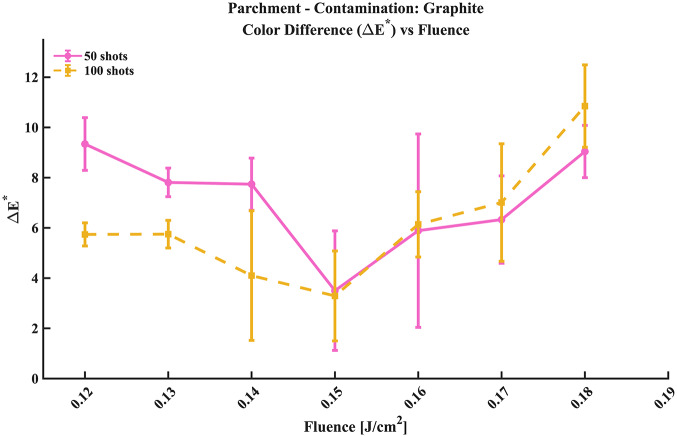
Color difference (ΔE*) values measured on the parchment sample contaminated with graphite after fs-laser cleaning, as a function of fluence for 50 and 100 laser shots. Error bars represent standard deviations from triplicate measurements.

The parchment sample exhibited notably lower reflectance variability than the leather substrates, which aligns with their smoother, less porous surface morphology. As shown in Fig. 11 and Table 3, ΔE* values followed a more coherent and predictable trend across both 50- and 100-shot conditions, highlighting the reduced impact of microtopographic irregularities.

**Table. 3:** Total color difference ΔE* for the fs-laser cleaned parchment sample at varying fluence and shot numbers

Sample name	Fluence [J/cm^2^]	ΔE^*^ (50 shots)	ΔE^*^ (100 shots)
Parchment	0.12	9.34 ± 1.05	5.74 ± 0.46
	0.13	7.81 ± 0.57	5.75 ± 0.55
	0.14	7.74 ± 1.04	4.10 ± 2.58
	0.15	3.50 ± 2.38	3.29 ± 1.79
	0.16	5.89 ± 3.85	6.14 ± 1.30
	0.17	6.33 ± 1.74	7.01 ± 2.34
	0.18	9.04 ± 1.04	10.85 ± 1.64

Values are reported as ΔE* ± propagated uncertainty (*σ*_ΔE*_).

Under 50-shot exposures, ΔE* steadily decreased from 9.34 ± 1.05 at 0.12 J/cm^2^ to a minimum of 3.50 ± 2.38 at 0.15 J/cm^2^, before increasing again to 9.04 ± 1.04 at 0.18 J/cm^2^. A similar pattern was observed with 100-shot cleaning, where ΔE* dropped to 3.29 ± 1.79 at 0.15 J/cm^2^ and then rose sharply to 10.85 ± 1.64 at 0.18 J/cm^2^. This consistent dip around 0.15 J/cm^2^ suggests an optimal fluence window in which contamination is effectively removed without compromising the substrate’s visual or structural integrity.

Despite parchment’s uniform morphology, the increase in ΔE* values beyond 0.15 J/cm^2^ indicates that higher fluence levels can still lead to optical or structural alteration. Because no complementary structural or chemical diagnostics were applied here, the underlying mechanism cannot be assigned unambiguously; however, the trend indicates a narrowing processing window at higher fluence and underscores the need for careful parameter control to avoid unintended changes. This demonstrates that even substrates with minimal surface roughness require careful control of laser-cleaning parameters to avoid unintended changes.

None of the tested conditions for Orange colored leather, Havana colored leather, or parchment achieved ΔE* values below 2.0, the commonly accepted visibility threshold for the human eye. This suggests that residual contamination likely remained trapped within microscopic recesses, or that reflectance was altered due to partial ablation of exposed fibers. These interpretations align with the porosity analysis results, which revealed that Orange and Havana colored leather surfaces exhibit notable porosity and microstructural heterogeneity. In contrast, parchment displayed negligible porosity, as confirmed by optical microscopy, yet its microstructure still exhibited subtle undulations that could influence reflectance measurements. Overall, such surface characteristics contribute to the observed variability in colorimetric outcomes, highlighting that, regardless of parameter optimization, the complex interaction between the laser and heterogeneous or micro-irregular surfaces imposes inherent limitations on achieving completely uniform cleaning results without altering the original substrate.

When examining the colorimetric data in more detail, it becomes clear that for each sample, both 50-shot and 100-shot conditions yielded ΔE* values that approached the commonly accepted threshold, despite not falling below it:

- Orange colored leather reached 2.56 ± 0.89 at 0.14 J/cm^2^ (50 shots) and 2.63 ± 2.26 at 0.13 J/cm^2^ (100 shots),
- Havana colored leather showed 2.12 ± 0.91 at 0.1114 J/cm^2^ (50 shots) and 2.41 ± 1.71 at 0.0995 J/cm^2^ (100 shots),
- Parchment achieved 3.29 ± 1.79 at 0.15 J/cm^2^ (100 shots).

These values represent averages across the entire measurement spot, including both elevated zones and recessed micro-areas. It is likely that if measurements had been confined to well-cleaned, elevated surface regions—where laser interaction is most direct and effective—ΔE* values would have fallen below 2.0 for all substrates. At the macroscopic scale, such regions may appear visually clean and homogeneous. However, contaminants trapped within fibrous pores or topographic recesses would remain undetected, leading to an overestimation of cleaning efficacy.

In colorimetric analysis, it is also important to evaluate the component-wise CIE L*a*b* coordinates differences (ΔL*, Δa*, Δb*), as they provide complementary information on whether the changes arise primarily from brightness (ΔL*), from red–green balance (Δa*), or from yellow–blue balance (Δb*). While ΔE* summarizes the overall color difference in a single value, it does not indicate which component dominates the variation. In heterogeneous substrates such as leather, this distinction is particularly relevant because surface roughness and porosity can affect brightness and chromaticity differently across the irradiated area.

To illustrate this point, Table 4 reports CIE L*a*b* coordinate differences, ΔL*, Δa*, and Δb*, at the irradiation conditions that produced the lowest ΔE* (closest to the perceptibility threshold). These examples highlight how the three coordinates contribute differently to the overall ΔE* and reveal the role of substrate morphology in shaping the observed variability.

**Table. 4:** CIE L*a*b* coordinate differences (ΔL*,Δa*, Δb*) are reported as mean ± standard deviation, while the total color difference (ΔE*) is reported as value ± propagated uncertainty

Sample name & fluence	ΔE*	ΔL*	Δa*	Δb*
Orange colored, 0.13 J/cm^2^	2.63 ± 2.26	1.40 ± 1.10	−2.07 ± 1.67	1.03 ± 1.97
Havana colored, 0.0995 J/cm^2^	2.41 ± 1.71	−2.17 ± 1.51	0.30 ± 1.50	1.00 ± 2.46
Parchment, 0.15 J/cm^2^	3.29 ± 1.79	−3.13 ± 1.83	−0.37 ± 0.26	−0.93 ± 1.35

All values correspond to 100-shot exposures.

As shown in Table 4, the analysis of individual CIE L*a*b* coordinate differences provides valuable insight into the origins of the observed color changes. For Orange colored leather at 0.13 J/cm^2^,100 shots, ΔE* was relatively low (2.63 ± 2.26), yet the underlying coordinate differences indicated a brightening trend (ΔL* positive) combined with a shift toward green (Δa* negative), consistent with morphology-driven reflectance variability and non-uniform residual soiling across the treated region. At the same time, Δb* showed substantial dispersion across measurement spots, indicating locally variable yellow–blue shifts relative to the reference. These variations are consistent with the spatial heterogeneity of the surface: measurements collected from protruding peaks, intermediate zones, and recessed pores inherently capture different local cleaning efficiencies. Havana colored leather at 0.0995 J/cm^2^, 100 shots also approached the perceptibility threshold (ΔE* = 2.41 ± 1.71), but the decrease in lightness (ΔL* negative) together with wide fluctuations in Δa* and Δb* again reflects heterogeneous outcomes across zones of different topography. In contrast, parchment at 0.15 J/cm^2^, 100 shots exhibited a somewhat higher ΔE* (3.29 ± 1.79), but its chromatic coordinates remained comparatively stable, with the change dominated by a consistent darkening (ΔL* strongly negative). These examples demonstrate that leather substrates exhibit pronounced coordinate-level variability driven by porosity and micro-topography, whereas parchment shows more uniform responses. At higher fluence values, where ΔE* values increase, such fluctuations in ΔL*, Δa*, and Δb* become even more pronounced, reinforcing the importance of evaluating coordinate-level changes alongside total color difference.

This finding highlights the significance of high-resolution, spatially localized reflectance measurements in assessing the performance of fs-laser cleaning on porous and morphologically heterogeneous organic heritage materials. Area-averaged colorimetry, although informative, may obscure critical microstructural variations and lead to misleading conclusions about cleaning success. For conservation tasks where visual consistency and material integrity are paramount, localized optical evaluation is crucial to accurately assess cleaning outcomes and to prevent long-term preservation risks that may be hidden beneath apparent surface uniformity.

Although post-cleaning reflectance measurements revealed variability across the leather surface, one might question whether targeting smaller, well-cleaned regions using a reduced laser spot size could improve measurement consistency or even cleaning precision. In theory, focusing the laser beam to smaller spot sizes, approaching the diffraction limit, could enable more localized cleaning and better alignment with the heterogeneous pore distribution observed on the leather surface. The minimum achievable spot size is ultimately governed by the diffraction limit, which is dependent on the laser wavelength and the numerical aperture (NA) of the focusing optics. For a laser operating at 1030 nm, the Rayleigh criterion suggests that spot diameters in the range of 2–3 µm are physically attainable under ideal conditions using high-NA objectives. However, such a reduction in beam size poses several challenges in the context of conservation science.

First, as the spot size decreases, the area that can be treated per unit time shrinks significantly, leading to a dramatic increase in cleaning duration. For example, reducing the spot from 500 µm to 5 µm corresponds to a 10,000-fold decrease in beam area. While the fluence (J/cm^2^) can theoretically be kept constant by proportionally reducing the pulse energy, practical limitations may emerge when operating at very low pulse energies, including reduced attenuation headroom, pulse-to-pulse stability constraints in certain operating regimes, and increased sensitivity to calibration uncertainty. Importantly, nonlinear effects are governed by peak intensity rather than pulse energy alone; therefore, if the fluence at the surface is truly kept constant, tight focusing by itself should not inherently increase nonlinear interaction pathways. Nevertheless, under extreme focusing conditions, small variations in beam quality and local field distribution can become more consequential, which may reduce process robustness on rough, heterogeneous surfaces.

Second, extreme focusing conditions typically imply a shorter working distance and a smaller depth of focus. This makes the process more sensitive to surface micro-relief, height variations, and local tilts, which are pronounced in porous leather. Maintaining a consistent focus condition over an extended treatment area, therefore, becomes significantly more difficult, and the delivered interaction conditions may vary locally even when nominal parameters are unchanged.

Third, if one aims to treat extended areas using micrometer-scale spots, the scanning burden increases substantially. For a fixed treated area and comparable overlap strategy, the number of scan positions scales approximately with the inverse of the spot area, so moving from sub-millimeter to micrometer-scale spots can increase the required number of positions by orders of magnitude. In practice, this translates into a strong throughput penalty irrespective of the repetition rate used, and it motivates the need to balance spatial resolution against treatment time and operational practicality in conservation settings.

Overall, although reducing the spot size offers theoretical advantages for selective cleaning and localized reflectance probing, practical constraints related to working distance, depth of focus, process robustness, and processing time render this approach less suitable for bulk laser cleaning of historical collagen-based materials. Future work could investigate strategies that improve robustness against micro-relief while maintaining throughput, including optical approaches that reduce sensitivity to local topography.

## Discussion

In this study, femtosecond laser cleaning reduced visible graphite contamination on historical vegetable-tanned goat leather; however, the colorimetric results exhibited substantial point-to-point dispersion, driven by surface porosity and microrelief. In contrast, the parchment control, characterized by negligible porosity but pronounced microtopography, displayed more coherent colorimetric trends, reinforcing the notion that surface morphology strongly governs the consistency of optical assessment on collagen-based substrates.

Our evaluation relied on micro-spot color measurements with a sampling area smaller than the ~500 µm laser beam footprint, revealing substantial variability across replicate readings. Even under identical cleaning conditions, ΔE* values spanned wide ranges, with standard deviations often exceeding 1.0 in several cases. This dispersion is consistent with non-uniform laser–surface interaction on porous leather, where protruding regions can experience a higher effective local fluence and be cleaned more strongly, while recessed micro-features such as pores and crevices can remain partially shielded and retain residues. This spatial mismatch introduces a practical challenge: ΔE* values from point-based reflectance measurements may not fully represent overall cleaning effectiveness or safety across the treated zone. For instance, a low ΔE* can reflect an average of well-cleaned protrusions and still-soiled recessed regions rather than a uniformly successful treatment.

Beyond overall porosity, the pore-size distribution is also likely to contribute. The coexistence of small and large pores produces complex surface micro-relief that can enhance geometric shielding and contribute to locally incomplete cleaning, thereby amplifying reflectance variability and limiting the uniformity of the apparent cleaning outcome. These observations highlight that ΔE* alone cannot fully capture cleaning efficacy on morphologically heterogeneous leather surfaces. For conservation practice, this implies that colorimetry should be interpreted explicitly in relation to surface morphology and used as one component of a multi-diagnostic assessment rather than as a stand-alone success criterion.

Finally, while ultra-localized laser targeting using strongly reduced spot sizes could, in principle, improve selectivity for specific micro-areas, the associated constraints in working distance, depth of focus, process robustness on micro-relief surfaces, and throughput make it impractical for bulk cleaning of historical leather. A more realistic strategy is to acknowledge the intrinsic limitations of optical metrics on porous substrates, to report cleaning conditions in a reproducible fluence-based framework, and to integrate spatially localized diagnostics when defining safe and effective processing windows for heritage leather and related collagen-based materials.

## Data Availability

The datasets generated and/or analyzed during the current study are not publicly available because they consist of experimental measurement files and microscopy/image datasets associated with the present study, but are available from the corresponding author on reasonable request.
